# ASO Author Reflections: Residual Disease in the Breast Following Neoadjuvant Chemotherapy Does Not Mandate Routine Post-Mastectomy Radiation Therapy/Regional Nodal Irradiation

**DOI:** 10.1245/s10434-020-09138-6

**Published:** 2020-09-12

**Authors:** Angelena Crown, Monica Morrow

**Affiliations:** 1 Breast Service, Department of Surgery, Memorial Sloan Kettering Cancer Center, New York, NY

## Past:

Postmastectomy radiation therapy (PMRT) and regional nodal irradiation (RNI) improve survival among patients who are at high risk of locoregional recurrence (LRR). While PMRT/RNI is routinely recommended to patients with nodal metastases, the benefit of PMRT/RNI in node negative patients remains poorly defined despite their inclusion in two randomized trials of PMRT/RNI.^1,2^ While data to inform decisions regarding PMRT/RNI following neoadjuvant chemotherapy (NAC) are limited, multiple studies, including the joint analysis of the National Surgical Adjuvant Breast and Bowel Project (NSABP) B-18 and B-27 trials, have identified absence of pathologic complete response (pCR) as a predictor of LRR.^3^

However, while residual disease is associated with increased LRR among patients with human epidermal growth factor receptor 2 (HER2) positive and triple negative (TN) tumors, patients with hormone receptor positive (HR+) tumors have low LRR rates regardless of NAC response.^4,5^ Whether the absolute risk of LRR is high enough to justify PMRT/RNI for node-negative patients with residual disease in the breast after NAC is uncertain.

## Present:

Using the prospective Memorial Sloan Kettering Cancer Center breast cancer database, we evaluated rates of LRR in a contemporary, unselected cohort of 227 women with cT1-T3N0 pN0 disease who failed to achieve pCR and identified high-risk features that warrant consideration of PMRT/RNI.^6^ Patients underwent lumpectomy with negative margins and whole breast radiation (n = 123, 54%) or mastectomy (n = 104, 46%); patients who received PMRT/RNI were excluded. Patients were divided into TN (n = 82, 36%), HER2+ (n = 73, 32%), and HR+/HER2- (n = 72, 32%) subtypes. Presence of LVI on final pathology was uncommon (n = 32, 14%).

After a median follow-up of 35 months (range 6–97), 9 patients experienced LRR (5 after mastectomy, 4 after lumpectomy), for a 3-year actuarial LRR rate of 5.9%. Six were regional recurrences and 3 were local recurrences. Median age of patients with LRR was 42 years (range 31–62) compared to 52 (range 28–87) for patients without LRR. TN patients had a 3-year actuarial LRR rate of 10.1% compared to 3.3% for HER2+ and 3.2% for HR+/HER2- patients. The 3-year actuarial LRR rate for patients with LVI was 11.9%.

## Future:

LRR in this unselected contemporary cohort of node-negative patients with residual disease in the breast was uncommon among HER2+ and HR+/HER2- patients, with 3-year actuarial rates ≤3% in the absence of PMRT/RNI compared to 10% for TN patients. While the low event rate precluded statistical analysis of clinicopathologic features associated with LRR, LRR following NAC appears to be influenced by multiple factors, likely including molecular subtype, presence of LVI, and age. Importantly, this study indicates that residual disease in the breast as a single risk factor does not confer an LRR risk >10% among node-negative patients and should not, in isolation, be considered an indication for routine PMRT/RNI. Future efforts to further refine patient selection for PMRT/RNI should consider the extent of residual disease in the breast and determine if the complete absence of response identifies a high-risk population independent of molecular subtype.
